# Quantification of effectiveness of bilateral and unilateral neuromodulation in the rat bladder rhythmic contraction model

**DOI:** 10.1186/1471-2490-13-34

**Published:** 2013-07-18

**Authors:** Xin Su, Angela Nickles, Dwight E Nelson

**Affiliations:** 1 Medtronic, Inc, Neuromodulation Research, 7000 Central Avenue NE, RCE470, Minneapolis, MN 55432, USA; 2 Medtronic, Inc, Physiology Research Laboratory, 11520 Yellow Pine St, Coon Rapids, MN 55448, USA

**Keywords:** Electrical stimulation, Bilateral, Bladder, Micturition, Spinal nerve

## Abstract

**Background:**

Using the isovolumetric bladder rhythmic contraction (BRC) model in anesthetized rats, we have quantified the responsiveness to unilateral and bilateral stimulation of the L6 spinal nerve (SN) and characterized the relationship between stimulus intensity and inhibition of the bladder micturition reflex.

**Methods:**

A wire electrode was placed under either one or both of the L6 SN roots. A cannula was placed into the bladder via the urethra and the urethra was ligated. Saline infusion induced BRC.

**Results:**

At motor threshold (T_mot_) intensity, SN stimulation of both roots (10 Hz) for 10 min reduced bladder contraction frequency from 0.63 ± 0.04 to 0.17 ± 0.09 contractions per min (26 ± 14% of baseline control; n = 10, p < 0.05). However, the same intensity of unilateral stimulation (n = 15) or sequential stimulation of both SNs (e.g. 5 min per side alternatively for a total of 10 min or 20 min) was less efficacious. The greater sensitivity to bilateral stimulation is not dependent upon precise bilateral timing of the stimulation pulses. Bilateral stimulation also produced both acute and prolonged- inhibition on bladder contractions in a stimulation intensity dependent fashion.

**Conclusions:**

Using the bladder rhythmic contraction model, bilateral stimulation was more effective than unilateral stimulation of the SN. Clinical testing should be conducted to further compare efficacies of unilateral and bilateral stimulation. Bilateral stimulation may allow the use of lower stimulation intensities to achieve higher efficacy for neurostimulation therapies on urinary tract control.

## Background

InterStim® Therapy, utilizing electrical stimulation of the sacral spinal nerve (SN, S3), is an established treatment for patients with overactive bladder [1]. The classical unilateral stimulation technique uses an electrode implanted in the S3 foramen and connected to an implanted pulse generator [2,3].

While unilateral stimulation has been compared to bilateral stimulation in several clinical trials, the results have been inconclusive. Bilateral stimulation, the simultaneous electrical stimulation of bilateral nerve roots, has been utilized and evaluated for efficacy using either an acute test [4,5] or chronic neuromodulation for over 1 year [6,7]. Scheepens and colleagues [4] reported that bilateral stimulation was superior to unilateral sacral neuromodulation in only 2 out of 25 patients. However the study was limited by its cross-over design using acute 4-day treatment with either bilateral or unilateral stimulation in the same individual with a 2-day recovery. In contrast, retrospective studies show that patients receiving bilateral stimulation are more likely to show a positive response to short term testing [5] and to have a significant symptomatic improvement in response to chronic implantation [7]. A complete understanding of unilateral and bilateral neuromodulation would be greatly facilitated by both preclinical and early-phase clinical work that better defines optimal stimulation parameters, stimulation timing for both unilateral and bilateral presentation of the stimulations.

In preclinical models in the cat [8,9] and pig [10], bilateral stimulation has been reported to produce a stronger bladder inhibition than unilateral stimulation. But stimulation parameters (such as current intensity or stimulation frequency) have not been optimized systemically and efficacies have not been compared quantitatively.

We have developed a preclinical model for routine screening of neurostimulation parameters and targets in the rat [11]. Our previous work demonstrated an intensity-dependent effect of stimulation on bladder inhibition in response to bilateral electrical stimulation of the L6 SN using a single wire electrode and single current control. In those experiments, stimulation pulses delivered to the two nerve roots are accurately time-matched, but current intensities were not individually controlled bilaterally depending on the functional impedance at each implantation site. In the present study, we specifically tested and controlled the delivery to both sides independently allowing a more complete characterization of stimulation thresholds for unilateral and bilateral stimulation of the SN. We quantitatively compared the efficacy of unilateral versus bilateral stimulation of the L6 SN on reflex bladder contractions and characterized the relationship between stimulus intensity and inhibition of the bladder micturition reflex. Current intensities between the two sides were either same (balanced) or different (unbalanced) relative to motor threshold (T_mot_). The effects of bladder micturition responses to bilateral neuromodulation using either pulses that are precisely time-matched or time-mismatched were also compared.

## Methods

Female Sprague–Dawley rats (200–300 g, n = 159) were anesthetized with urethane (i.p., 1.2 g/kg, 200 mg/ml in saline, Sigma-Aldrich, St. Louis, MO). Anesthetized rats were maintained at 37°C with a heating pad and were euthanized by CO_2_ asphyxia upon completion of experiments. The experimental protocols were approved by the Institutional Animal Care and Use Committee of Medtronic and Non-clinical Research Board of Medtronic (Minneapolis, MN).

**Figure 1:**
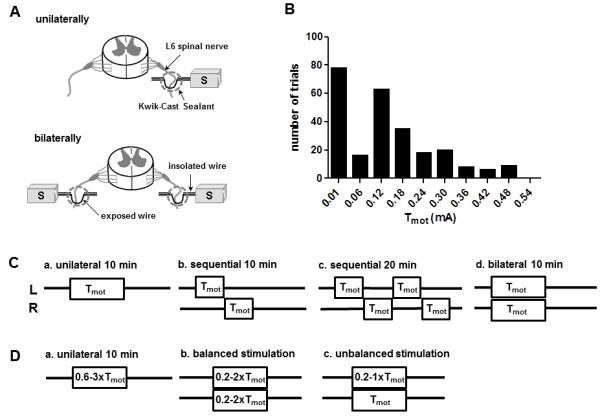
**Experimental model for spinal nerve stimulation. A.** Experimental setup and location of unilateral and bilateral electrode implantations. **B.** Histogram of motor threshold (T_mot_) to spinal nerve stimulation. **C** and **D**. Schematic drawings of the stimulation trials of unilateral or bilateral neuromodulation at T_mot_ intensity **(C)** and intensity dependent effects of spinal nerve stimulations **(D)**. Unilateral stimulation was delivered on the left (L) side of the spinal nerve and bilateral stimulation was applied simultaneously on both left and right (R) side of the nerve.

A cannula (PE50, inside diameter, 0.53 mm; outside diameter, 0.97 mm) was inserted into the bladder via the urethra, and secured with a suture tie for intravesical pressure recording and saline infusion. The L6 nerve trunks were localized caudal and medial to the sacroiliac junction. Unilateral stimulation was applied via one of the stainless steel wire electrodes (40-guage, Cooner Wire Co., Chatsworth, CA), placed under the left side of the L6 SN. Bilateral stimulation was produced using two electrodes, placed under both sides of the L6 SN (Figure 1A). The wire electrode(s) were positioned, secured with silicone adhesive, and connected to a Grass S88 stimulator (Grass Medical Instruments), through stimulus isolation unit(s) (SIU-BI, Grass Medical Instruments). A needle electrode under the skin of the tail served as the ground.

**Figure 2:**
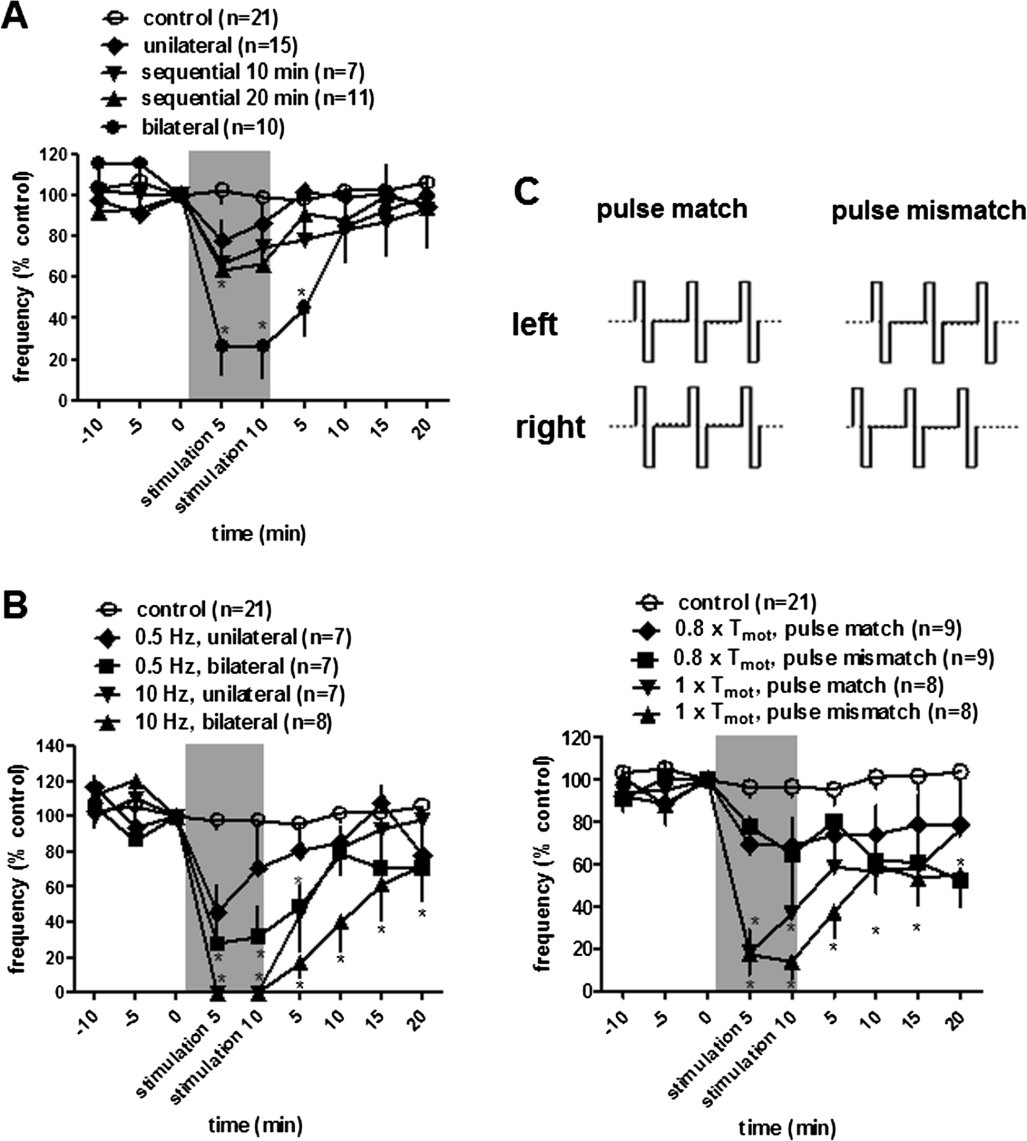
**Time course response of frequency of the bladder rhythmic contraction to unilateral and bilateral spinal nerve stimulation (10 Hz, pulse width 0.1 ms) at motor threshold intensity (A), a supra-threshold intensity of 0.6 mA (B) or bilateral 10 Hz neuromodulation in pulse matched or pulse mismatched fashion (C).** Schematic drawings of the stimulation waveform of pulse match and pulse mismatch are shown in C. There is no delay or a 50-ms delay between right and left pulses in pulse match or pulse mismatch, respectively. Responses are represented as a percentage of pretreatment values (% control), where the baseline response before stimulation is defined as 100%. * p < 0.05, vs control without stimulation; repeated measures ANOVA, Bonferroni post test. Shaded areas are responses during electrical stimulation.

Electrical stimulation of the SN evoked side-specific hind-toe twitches and/or pelvic floor muscle contraction [11-13]. In each rat, the threshold current (T_mot_) was defined as the lowest current required to evoke the first, barely observable, muscle contraction. For bilateral stimulation, the T_mot_ was measured on each side separately, to allow for potential differences between left and right nerve roots. Simultaneous stimulation of both roots at T_mot_ intensities did not produce an observable difference in muscle contractions compared to unilateral stimulation. Biphasic pulses (pulse width 0.1 ms) of different intensities (0.2 × T_mot_ – 3 × T_mot_, or 0.6 mA) were used to stimulate the SN. The intensity-dependent response of the bladder micturition reflex to SN neuromodulation was characterized using either bilateral stimulation or unilateral stimulation (Figure 1C and 1D). Current intensities between the two sides were either same (balanced) or different (unbalanced) relative to T_mot_ (Figure 1D). The effects of bladder micturition responses to bilateral neuromodulation using either pulses that are precisely time-matched or time-mismatched were also compared (Figure 2C). Stimulation in this study was applied at a fixed frequency of 10 Hz, which has been shown to be optimal for inhibition of bladder contractions by both low and high intensity stimulation [11,12].

To induce bladder rhythmic contractions (BRC), saline was infused into the bladder via the syringe pump (50 μL or 10 μL per min) until 3–5 consecutive contractions were established. At this time, the saline infusion was terminated. Each trial of recording lasted for 45 minutes including 15 min control, 10 min nerve stimulation and 20 min post stimulation. Two trials of the testing were performed with a random stimulation parameter in some rats. The bladder was emptied after finishing the first trial and BRC was re-established by saline infusion. The 2nd stimulation was applied at least 40 min after the 1st stimulation. A total of 241 trials were studied in 159 rats.

### Data analyses

SN stimulation did not reduce the amplitude of bladder contractions [11,13], therefore only effects on frequency/interval of BRC were studied. Data were calculated in 5 min bins, each having three control periods, two periods during stimulation, and four periods after stimulation. In addition, data were also normalized according to the mean response during the last 5 minutes prior to stimulation to compare response sensitivities.

All data are expressed as mean ± SEM. Student t-test (P < 0.05) was utilized to compare means of responses in the same treatment group or among the different groups. Time course for the BRC response to SN stimulation was analyzed using repeated measures ANOVA (Prism 5, GraphPad Software, Inc., San Diego, CA). Bonferroni post-hoc test was used to determine the statistical significance between different time points.

## Results

The motor threshold current (T_mot_) was 0.14 ± 0.01 mA (n = 253; range: 0.01 – 0.5 mA; 95% confidence interval (CI): 0.12 – 0.15 mA, Figure 1B).

There was no significant change in BRC during a 45 min recording if electrical stimulation was not applied (n = 21).

**Figure 3:**
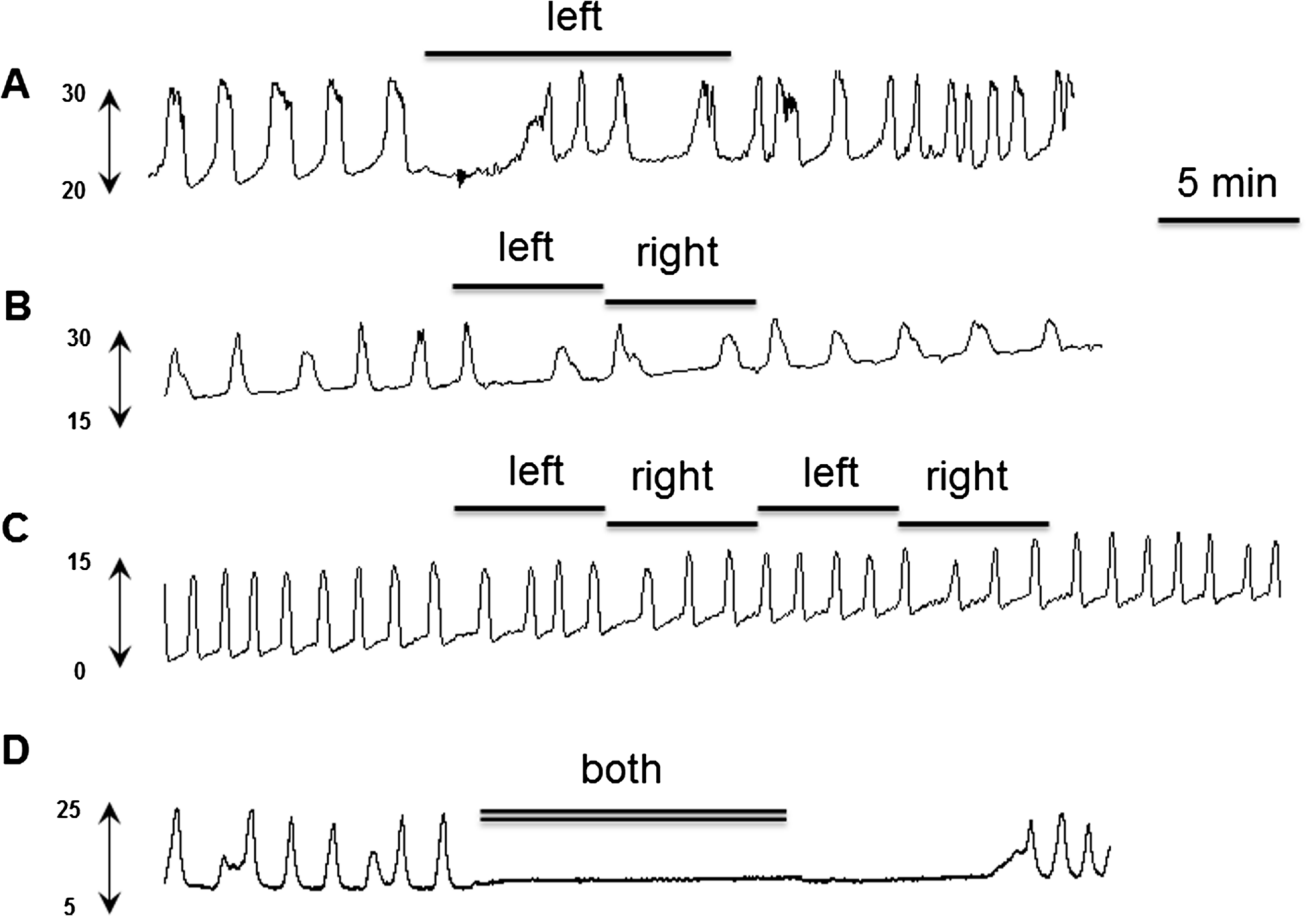
**Typical experimental records showing the bladder rhythmic contraction (mmHg) to unilateral stimulation at motor threshold intensity (10 Hz, pulse width 0.1 ms) of the left spinal nerve (SN, A) for 10 min, sequential, alternative 5-min stimulation of left SN and right SN for 10 min (B), sequential, alternative 5-min stimulation for 20 min (C) and bilateral electrical stimulation for 10 min.** Horizontal bars indicate duration of spinal nerve stimulation. Unilateral neuromodulation **(A)**, 10 min sequential stimulation **(B)** or 20 min sequential stimulation **(C)** produced little to no reduction of the frequency of bladder contractions during electrical stimulation. Bilateral stimulation eliminated bladder contractions; after termination of the stimulus, the effect persisted for a few minutes. When bladder contractions recurred, their amplitude was comparable to that before stimulation **(D)**.

Figure 2 summarizes BRC responses to unilateral and bilateral SN stimulation and Figure 3 depicts individual responses to stimulation that are representative of the overall data. Repeated measures ANOVA demonstrates that a significant inhibition of BRC frequency is produced by bilateral SN stimulation at T_mot_ intensity (p < 0.05, vs. control, n = 21, Figure 2A, Table 1). Maximal inhibition to bilateral stimulation appeared during stimulation and the effect persisted for 5 min after termination of the stimulus. This effect is significantly greater than that produced by unilateral SN stimulation (p < 0.05, repeated measures ANOVA). There is no correlation between the pre-stimulation contraction frequency and the response to bilateral stimulation (p = 0.27, R square = 0.15, correlation). Sequential, alternative stimulation of the SN for 20 min (the total duration on each nerve root was equivalent to 10 min bilateral stimulation) produced mild reduction of the frequency of bladder contractions during electrical stimulation (p < 0.05, repeated measures ANOVA, Figure 2A, Table 1). As a comparison in Figure 2A, 20 min stimulation period was scaled to fit into the 10 min stimulation period, the “stimulation 5” value is the mean of the first 5 min left and subsequent right SN stimulations and “stimulation 10” value is the mean of the second 5 min left and subsequent right SN stimulations. Analysis revealed no significant differences in bladder contraction frequency in response to unilateral stimulation and 10 min sequential stimulation, respectively (p > 0.05).

**Table 1:** Contraction frequency (contractions per min) to spinal nerve Stimulation

	**Before**	**Treatment**	**10 min post**	**20 min post**
without stimulation	0.64 ± 0.06	0.62 ± 0.05	0.63 ± 0.07	0.65 ± 0.07
10 Hz, T_mot_, unilateral	0.58 ± 0.05	0.51 ± 0.07	0.63 ± 0.07	0.62 ± 0.08
sequential 10 min	0.67 ± 0.04	0.50 ± 0.11	0.60 ± 0.12	0.64 ± 0.11
sequential 20 min	0.55 ± 0.05	0.38 ± 0.10 * +	0.53 ± 0.07	0.56 ± 0.07
10 Hz, T_mot_, bilateral	0.63 ± 0.04	0.17 ± 0.09 * + #	0.37 ± 0.09 * + #	0.66 ± 0.14
0.5 Hz, 0.6 mA, unilateral	0.57 ± 0.09	0.30 ± 0.09 * +	0.47 ± 0.10	0.53 ± 0.10
0.5 Hz, 0.6 mA, bilateral	0.85 ± 0.06	0.20 ± 0.08 * +	0.50 ± 0.08	0.60 ± 0.15
10 Hz, 0.6 mA, unilateral	0.75 ± 0.06	0 ± 0 * +	0.43 ± 0.09	0.66 ± 0.06
10 Hz, 0.6 mA, bilateral	0.72 ± 0.10	0 ± 0 * +	0.20 ± 0.07 * +	0.49 ± 0.15
0.8 × T_mot_, pulse match	0.47 ± 0.03	0.32 ± 0.05	0.36 ± 0.04 +	0.37 ± 0.06 +
0.8 × T_mot_, pulse mismatch	0.56 ± 0.06	0.38 ± 0.07 +	0.36 ± 0.07	0.29 ± 0.06 +
1 × T_mot_, pulse match	0.42 ± 0.04	0.14 ± 0.06 * +	0.26 ± 0.06 +	0.30 ± 0.07 +
1 × T_mot_, pulse mismatch	0.54 ± 0.10	0.09 ± 0.05 * +	0.22 ± 0.05 +	0.23 ± 0.03 +

* P < 0.05, paired student’s t test for intragroup comparison to “before treatment”.

+ P < 0.05, unpaired student’s t test for intergroup comparison to “without stimulation”.

# P < 0.05, unpaired student’s t test for intergroup comparison to “unilateral stimulation”.

Figure 2B shows the time course of the mean responses of BRC frequency to unilateral and bilateral stimulation at a supra-threshold intensity of 0.6 mA, which has been shown to induce maximal inhibition of bladder contractions [11]. Maximal inhibition appeared during stimulation in all groups, but only the inhibition of BRC produced by bilateral SN stimulation was sustained for 20 min post-stimulation (prolonged inhibition, p < 0.05, repeated measures ANOVA).

Either unilateral or bilateral stimulation (0.6 mA) at 10 Hz completely abolished bladder contractions. Using 0.5 Hz stimuli at the same intensity, which produces about ~50% bladder inhibitory response [11], unilateral and bilateral stimulation produced an inhibition on bladder contraction frequency from 0.57 ± 0.09 and 0.85 ± 0.06 to 0.30 ± 0.09 (58% of control, n = 7) and 0.20 ± 0.08 (29% of control, n = 7) respectively, with a trend toward greater inhibition by bilateral stimulation (p = 0.25, Figure 2B, Table 1).

To determine whether the inhibition of BRC requires precisely phase locking of stimulation pulses on each side of the SN, bilateral stimulation was applied using pulse matched (no delay between right and left pulses) and pulse mismatched stimulus trains (50-ms delay, Figure 2C). Ten Hz pulse matched stimulation at 0.8 × T_mot_ or 1 × T_mot_ showed an equal effect as pulse mismatched (Table 1).

**Figure 4:**
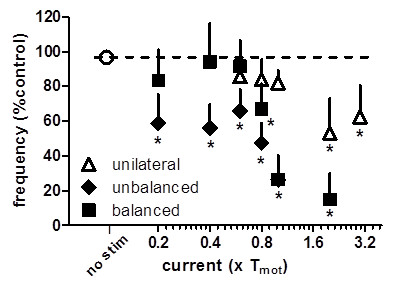
**Intensity dependent effects of unilateral, unbalanced bilateral and balanced bilateral spinal nerve (SN) stimulations on frequency of bladder contractions during electrical stimulation.** X-axis is the varied current intensity relative to multiples of motor threshold (T_mot_) stimulation. Balanced stimulation had equal intensities on left and right SN. Unbalanced stimulation was delivered at a fixed intensity of T_mot_ on one side while the current intensity was varied on the SN of the other side. The response of contraction frequency is the mean value as a percentage of pretreatment values (% control) during stimulation. *p < 0.05, vs control without stimulation, Student’s t-test.

Figure 4 shows the effect of SN stimulation at 10 Hz on BRC at different stimulation intensities. The inhibition of the contraction frequency was greater as the current intensity increased. The smallest intensity of bilateral stimulation that produced a statistically significant bladder inhibition (p < 0.05, vs. without stimulation, n = 21, Student’s t test) was 0.8 × T_mot_ for balanced stimulation. This stimulation decreased the frequency of bladder contractions to 67 ± 14% of pretreatment value, or 0.2 × T_mot_ for unbalanced stimulation decreasing the frequency of bladder contractions to 58 ± 16% of pretreatment value (n = 6). The minimal effective intensity of unilateral stimulation was 2 × T_mot_, decreasing the frequency of bladder contractions to 53 ± 20% pretreatment value (n = 12).

## Discussion

Compared with unilateral stimulation, bilateral stimulation produced attenuation of bladder contractions at lower stimulation levels. The effective stimulation thresholds were 0.8 × T_mot_. Unilateral stimulation was less efficacious, with a current threshold of 2 × T_mot_. Our data does not show a clear difference in the results of bilateral stimulation using either equal (*balanced*) or unequal (*unbalanced*) stimulation intensity on left and right SN.

The rat SN is composed of nerve fibers emerging from the pelvic nerve, the pudendal nerve, and the somatic nerve bundles. The somatic bundles derive from the sciatic nerve, including the tibial nerve and others [14-16]. It is possible that stimulation at low current intensity activates mainly the large somatic afferent fibers to produce bladder inhibitory effects [13]. Electrical stimulation at high current intensity activates small fibers which may elicit reflex bladder inhibition via the pelvic [17,18] and dorsal genital [13] nerves. In any case, high intensity stimulation of pelvic nerve and dorsal genital nerve may not be required for a bladder inhibitory effect since bilateral neuromodulation is efficacious at low current intensity.

In cats the inhibitory mechanism involved in the antidromic stimulation of pelvic motor neurons has been suggested to be activation of the inhibitory interneurons by recurrent collaterals of pelvic motor nerves, inhibiting the contralateral pelvic motoneurons (recurrent inhibition) [18,19]. In rats, the hypothesized mechanisms of action of neuromodulation to low intensity stimulation appears to alter the transmission of sensory input from the bladder to the pontine micturition center, while the inhibitory effects on bladder contraction evoked by high intensity stimulation may be mediated additionally through efferent limb of the micturition reflex arc [11]. However, direct efferent activation or a short loop reflex triggered by low intensity nerve stimulation is not likely to be involved since bilateral stimulation with phase match or mismatched pulses at intensity = / < T_mot_ produced an equivalent bladder inhibitory response. One would assume that output signals would be synchronized if the perfectly matched pulses from right and left SN stimulation are transmitted directly. Therefore enhanced bladder inhibitory effects to bilateral stimulation could be attributed to a polysynaptic reflex at the spinal and supraspinal centers [20].

Sequential unilateral stimulation of left and right spinal nerves (10 min or 20 min, temporal summation), is not significantly more effective than stimulation of only one SN root. In the cat, stimulation of multiple segments of one sacral nerve was no more effective than unilateral stimulation [9]. Therefore, spatial summation, resulting from simultaneous stimulation of both nerve roots of the same spinal segment is required for producing effective neuromodulation of bladder activity at low stimulation intensity. The requirement for this spatial summation could be associated with the bilateral innervation of the urinary bladder [21-23].

Unilateral neuromodulation by stimulating one side of the SN may modulate micturition reflex via control of bilateral efferent outflow in the spinal cord as well as in the brain, but only be partially effective, either because it does not activate enough afferent fibers or because it does not allow new regulatory pathways to emerge. On the other hand, bilateral stimulation could activate more inhibitory interneurons in the spinal cord, or send stronger ascending signals to the brainstem.

Our finding that bilateral stimulation produced the largest inhibition of BRC frequency is consistent with other preclinical observations in the cat [8,9] and in the pig [10]. It is possible that unilateral stimulation at high current intensity (2 × T_mot_) activates more fibers and produces an inhibition of the BRC. Stimulation at low current intensity at T_mot_ may activate only large myelinated fibers [11] while high current intensity may stimulate unmyelinated C-fibers as well [24]. Clinically the stimulation amplitude of 1.3 times sensory threshold would cause patients to feel pain or discomfort [5], limiting the stimulation intensity for a useful therapeutic range. Accordingly, bilateral stimulation at T_mot_ or subthreshold intensities could widen the therapeutic window, allowing activation of more large myelinated fibers to produce bladder inhibition without activation of C-fiber afferents.

The current study using the isovolumetric RBC model in anesthetized animals has facilitated rapid screening of acute responses to bilateral stimulation. However, the present study, using only 10 min stimulation cannot directly address the sustained bladder inhibition seen in overactive bladder patients under continuous InterStim® therapy. In addition, the isovolumetric bladder contraction in an animal with a ligated urethra cannot result in voiding, and the neural modulation of this reflex may differ from that acting on the voiding reflex in a normal or pathologic bladder. Furthermore, a difference between anesthetized and conscious preparations cannot be excluded. Further experiments to measure effects on voiding frequency and volumes using conscious models will target the mechanisms by which neuromodulation acts to relieve the symptoms of overactive bladder.

## Conclusions

We demonstrate that bilateral stimulation produces a stronger inhibition of the bladder micturition reflex than unilateral or sequentially unilateral stimulation. The increased sensitivity to bilateral stimulation does not require precise pulse locking on each side of the SN. Potential application of bilateral stimulation using either equal (*balanced*) or unequal (*unbalanced*) intensities should be evaluated further in a clinical setting.

## Abbreviations

SN: Spinal nerve; Tmot: Motor threshold; BRC: Bladder rhythmic contraction.

## Competing interests

All authors are employees of Medtronic Inc. The research was supported by Medtronic Preclinical Research Funding.

## Authors’ contributions

Conception and design: XS. Acquisition of data: AN. Analysis and interpretation of data: XS, AN, DEN. Drafting the manuscript: XS. Revising it critically for important intellectual content: XS, DEN. Final approval of the version to be published: XS, AN, DEN. All authors read and approved the final manuscript.

## Pre-publication history

The pre-publication history for this paper can be accessed here:

http://www.biomedcentral.com/1471-2490/13/34/prepub
